# Pseudo-188D: Phage Protein Prediction Based on a Model of Pseudo-188D

**DOI:** 10.3389/fgene.2021.796327

**Published:** 2021-12-01

**Authors:** Xiaomei Gu, Lina Guo, Bo Liao, Qinghua Jiang

**Affiliations:** ^1^ Key Laboratory of Computational Science and Application of Hainan Province, Haikou, China; ^2^ Institute of Yangtze River Delta, University of Electronic Science and Technology of China, Haikou, China; ^3^ Key Laboratory of Data Science and Intelligence Education, Hainan Normal University, Ministry of Education, Haikou, China; ^4^ School of Mathematics and Statistics, Hainan Normal University, Haikou, China; ^5^ Beidahuang Industry Group General Hospital, Harbin, China

**Keywords:** model pseudo-188D, phage, stochastic gradient descent, dimensional disaster, digital characteristics

## Abstract

Phages have seriously affected the biochemical systems of the world, and not only are phages related to our health, but medical treatments for many cancers and skin infections are related to phages; therefore, this paper sought to identify phage proteins. In this paper, a Pseudo-188D model was established. The digital features of the phage were extracted by PseudoKNC, an appropriate vector was selected by the AdaBoost tool, and features were extracted by 188D. Then, the extracted digital features were combined together, and finally, the viral proteins of the phage were predicted by a stochastic gradient descent algorithm. Our model effect reached 93.4853%. To verify the stability of our model, we randomly selected 80% of the downloaded data to train the model and used the remaining 20% of the data to verify the robustness of our model.

## Introduction

The term bacteriophage is actually a generic name for viruses or microorganisms. Phage virus proteins can be either viruses that invade bacteria or genetic material. According to the literature, phages are the most diverse entities in the ocean and affect biochemical systems around the world (Jahn et al., 2019; Cheng et al., 2020). Phages also affect the development of anticancer drugs because phage fusion proteins can promote the amplification and manufacturing of combinatorial chemistry products and nanotechnology to be applied in clinical trials for cancer treatment (Petrenko and Jayanna, 2016; Cheng et al., 2018; Yu et al., 2021a; Yu et al., 2021b). Phages may also cause acute or chronic skin infections and, in severe cases, may lead to death in patients with multidrug resistance (Al-Wrafy et al., 2019). Phages may play a part in the spread of antibiotic resistance, and thorough investigation must determine whether they contain antibiotic-resistance genes (Lekunberri et al., 2017). Individual glycoside hydrolases have been identified in the phage virion, which may facilitate phage annotation during infection (Yuan and Gao, 2016). However, experimental methods for the identification of phage viral proteins are time-consuming, and the cost is very high. Additionally, the identification of phage viral proteins presents challenges due to the diversity of phages and their abundant physical functions, and databases for phage annotation are rare (Seguritan et al., 2012; Bhakta and Tsukahara, 2020; Cheng et al., 2021). This also increases our difficulties with phage identification, so novel methods are needed to overcome these shortcomings. Therefore, we must develop accurate and affordable methods to predict phage viruses. Meeting these requirements based on the sequence calculation method can overcome these difficulties (Zeng et al., 2017; Hong et al., 2019; Zou et al., 2019; Cai et al., 2020a; Cai et al., 2020b; Fu et al., 2020; Hasan et al., 2020; Hu et al., 2020; Li et al., 2020a; Meng et al., 2020; Naseer et al., 2020; Zhang et al., 2020a; Hu et al., 2021a; Hu et al., 2021b; Wang et al., 2021a), and using bioinformatics methods to identify phage proteins, such as analysing protein and amino acid composition (Wu et al., 2019; Xu et al., 2021a), can facilitate the extraction of features, combined with artificial neural networks (Chen et al., 2020) and the use of random forest (Ao et al., 2020; Chen et al., 2020; Zhang et al., 2020b; Ahmed et al., 2021) integrated indicators to identify protein phages (Zhang et al., 2015; Ba Lachandran et al., 2018; Wu and Yu, 2021). For the development of phage virus protein identification, we need not only an affordable identification method but also the accuracy to judge whether the method can be used.

**FIGURE 1:**
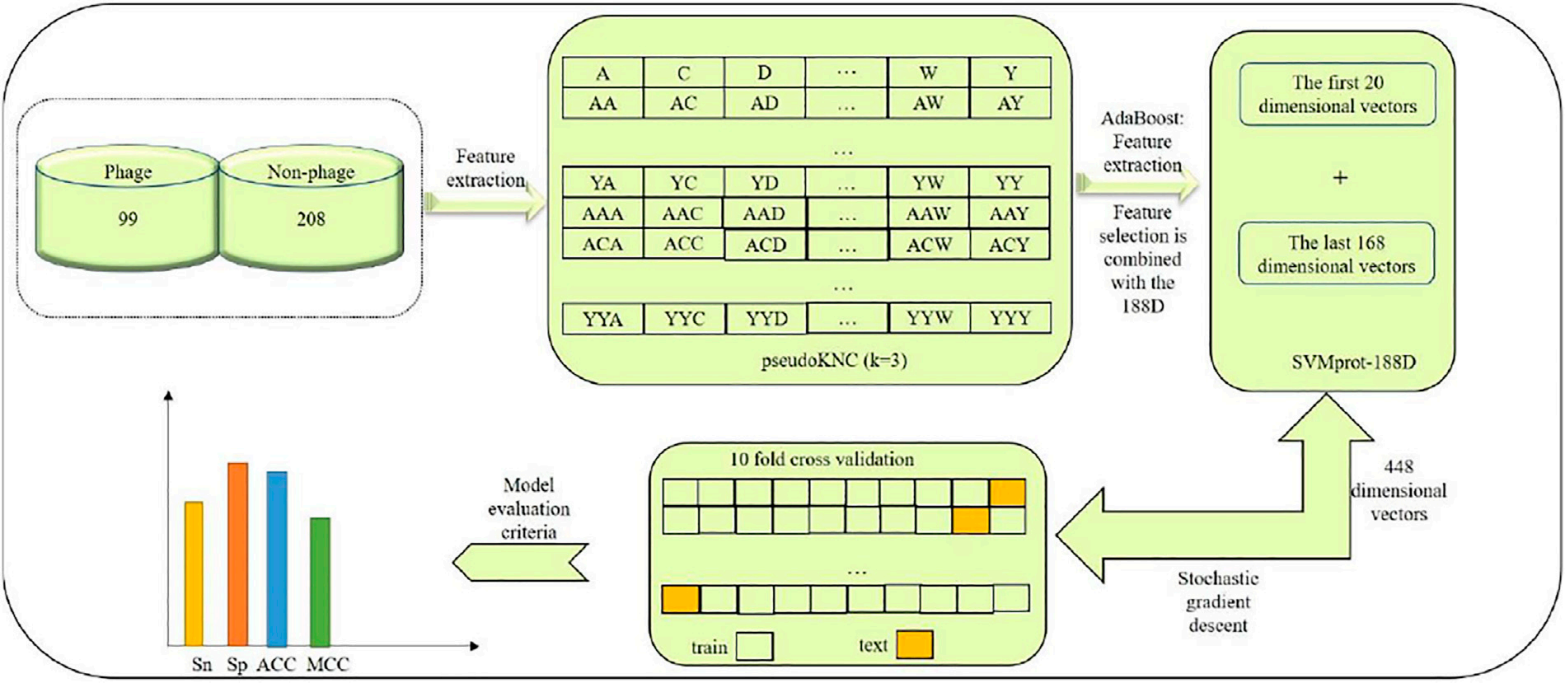
Establishing model Pseudo-188D process.

In this paper, we established a model of Pseudo-188D. The process of establishing this model involved first selecting suitable phage virus protein data and downloading the data from UniProt, which constituted our benchmark dataset, as our database for phage protein identification. Second, we used the pseudoKNC method to extract the digital characteristics of phages. In this process, we selected the appropriate value of ktuple (k) after tuning. Then, to reduce the impact of the dimensional disaster on the experimental results, the AdaBoost tool was used to select the appropriate vector. After selecting the appropriate feature vector, SVMprot-188D (188D) was used to extract the feature vector of the phage protein. After extracting the 188D feature, the features extracted by the two tools were combined. Finally, the random gradient descent (SGD) algorithm was used to predict phage proteins. To establish a model with stability and good robustness, we randomly selected 80% of the data as a test set to train the model and the remaining 20% of the data as a validation set to prove the stability of our model. At the same time, our model not only shows good stability and robustness but also very high accuracy. Readers can refer to Figure 1 for our model-building process, which clearly expresses our ideas.

## Materials and Methods

### Data

To better study phage proteins, we used data mainly from the literature (Meng et al., 2020). The data cited in this paper have been used in most studies for the identification of phage viral proteins because of their reliability and application to compare levels between different identifiers. The positive samples in the data were phages with viruses in subcellular positions, whereas the negative samples were nonphages. The sequences containing unrecognizable characters such as “Z”, “X”, “U”, and “B” were removed from the selected data. Finally, to avoid excessive homology of the data, redundant data were removed to ensure that the consistency between any data was not more than 40%, so our data included 99 phage virus protein-positive samples and 208 nonphage-negative samples. We will deposited the data at the website https://github.com/gxm123456/gxm.

### PseudoKNC

PseudoKNC is a kind of software for extracting the digital features of DNA, RNA, and protein, and the features extracted by this software are all digital features (Muhammod et al., 2019; Yang et al., 2020; Ao et al., 2021a; Cao et al., 2021; Jiao et al., 2021; Sheng et al., 2021). Because the characteristics of protein, DNA, and RNA sequences are different, the dimensions of the extracted features are also different (Zuo et al., 2017; Zheng et al., 2019; Ao et al., 2021b). When vis guaranteed, and when the extracted feature sequence is a DNA or RNA sequence, the extracted digital feature dimension is $\sum_{i=1}^{n}4^{i}$ ; when the extracted feature sequence is a protein sequence, the extracted digital feature dimension is $\sum_{i=1}^{n}20^{i}$ . For the value of k , how the k value affects the number and style of features we select will be introduced in detail below:

When the k value is set to 1, the extracted DNA and RNA sequence feature dimension is 4, the extracted protein sequence feature dimension is 20, and the extracted feature is X ;

When the k value is set to 2, the extracted DNA and RNA sequence feature dimension is 20, the extracted protein sequence feature dimension is 420, and the extracted feature is X , XX ;

When the k value is set to 3, the extracted DNA and RNA sequence feature dimension is 84, the extracted protein sequence feature dimension is 8,420, and the extracted feature is X , XX ,

Therefore, let us define X here: X stands for DNA, RNA, and protein sequences.

When the sequence is DNA, X={A,C,G,T} ;

When the sequence is RNA, X={A,C,G,U} ;

When the sequence is protein, X={A,C,D,E,F,G,H,I,K,L,M,N,P,Q,R,S,T,V,W,Y} .

**FIGURE 2:**
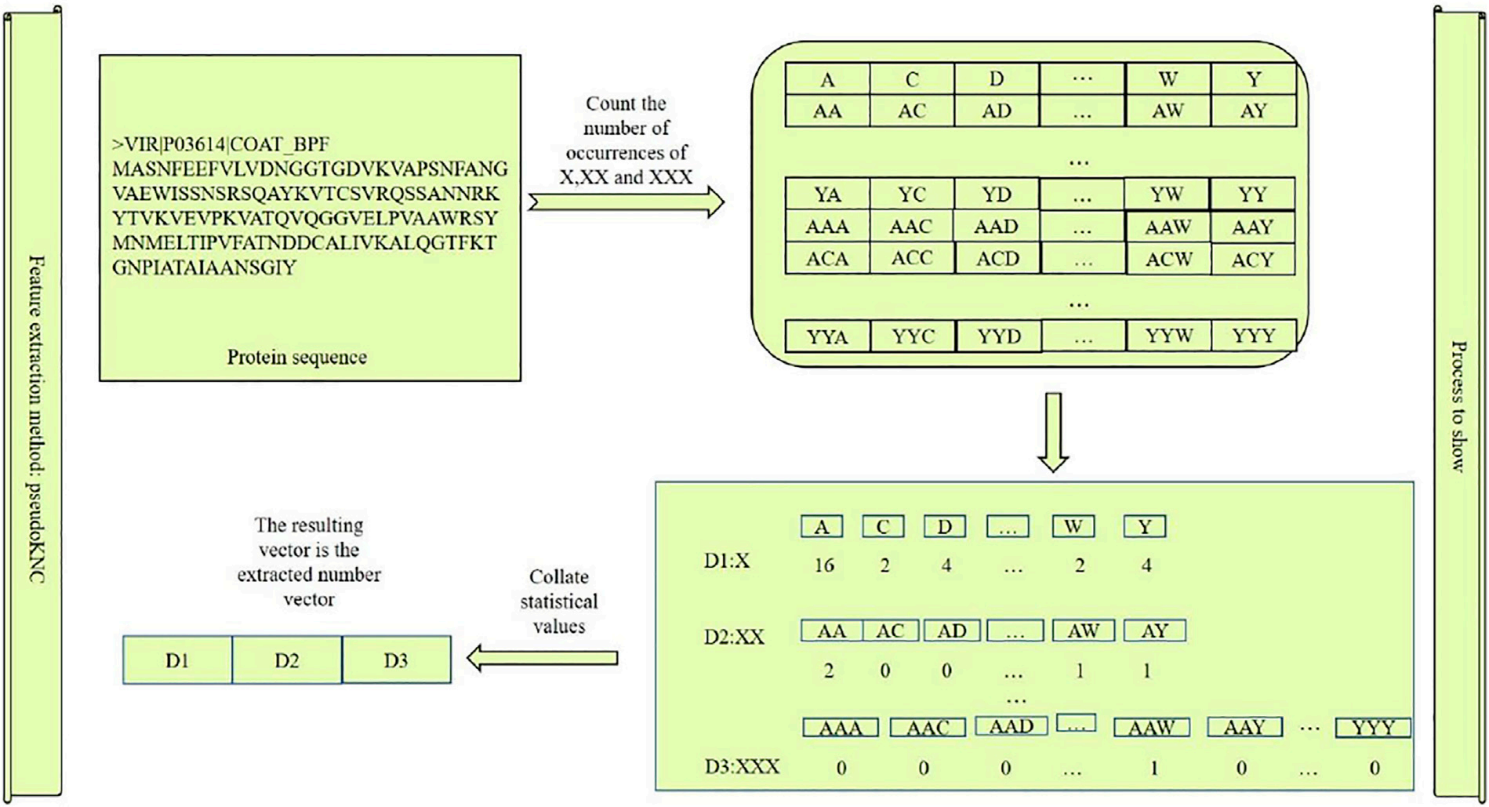
Extraction process of vector features by PseudoKNC.

Figure 2 can be used as an example to show the protein sequences we extracted. There are 8,420 features extracted by us. The first 20 feature styles are X , which simply form the protein sequence string arrangement, the middle 400 feature styles are XX , which form the protein sequence string arrangement in pairs, and the last 8,000 feature styles are XXX , which form the protein sequence string arrangement in three strings. Finally, the frequency of these permutations and combinations in the protein is counted, and the resulting vector is the feature we extracted.

### AdaBoost

The model AdaBoost is the SCRIT package used in Python, and to avoid any possible overfitting states, RNA and protein data are used as case studies, which can assess the generality of the model (Zhu et al., 2006; Cheng et al., 2016; Chen et al., 2019; Ramzan et al., 2021). After data selection is completed, n features with the best score are selected for training. The AdaBoost model only runs once and can select suitable features, which is more effective than other methods. The AdaBoost model incorporates different instance weight distributions into the impurity measurement and simultaneously increases the diversity of feature selection, so the adverse effects of multicollinearity features are reduced in the feature selection process.

### SVMProt-188D

This method can extract a total of 188 feature dimensions, so it is also called 188D (Li et al., 2020b). The 188D top 20 extraction dimension vectors were used to calculate the frequency of the arrangement for 20 kinds of natural amino acids (A, C, D, E, F, G, H, I, K, L, M, N, P, Q, R, S, T, V, W, Y) (Zheng et al., 2020). Mainly refer to Formula (1) for calculation:

$$\left(V_{1},V_{2},...,V_{20}\right)=\frac{N_{i}}{L}$$ (1)

In Formula (1), $N_{i}$ represents the number of the *i*th amino acid present in the protein sequence, and L represents the total number of amino acids contained in the sequence.

The next 168 features are associated with eight physicochemical properties, all represented by descriptors C (composed of amino acids), T (transition), and D (distribution). These three properties are made up of numbers, where C is composed of 3, representing the frequency of amino acids in a particular class; T is made up of three and represents the percentage of amino acids in the two different categories; D is made up of 15, representing the chain length ratios of the first, quarter, half and last amino acids in a given category, and then expanding the calculation by another hundred times. In this way, we extracted 168-dimensional features later:

(C+T+D)×8=168 (2)

This process encompasses the entire process for the extraction of 188 dimension features and the meaning of each feature.

### Stochastic Gradient Descent

The stochastic gradient descent algorithm determines an optimal path, and under the selection of this path, the optimal result is achieved by choosing the nearest shortcut. The main process of stochastic gradient descent is as follows:

$$h\left(\theta \right)=\theta_{0}x_{0}+\theta_{1}x_{1}+\theta_{2}x_{2}+...+\theta_{n}x_{n}=\sum \theta_{i}x_{i}$$ (3)

The stochastic gradient descent algorithm obtains the optimal data by taking partial derivatives of the coefficients many times. The θ value in Formula (3) decreases along the direction of the fastest gradient descent and finally obtains the optimal solution:

$$\begin{array}{l} \frac{\partial }{\partial \theta_{j}}J\left(\theta \right)=\frac{\partial }{\partial \theta_{j}}\frac{1}{2}{\left(h_{\theta }\left(x\right)-y\right)}^{2}\\ =2\cdot \frac{1}{2}\left(h_{\theta }\left(x\right)-y\right)\cdot \frac{\partial }{\partial \theta_{j}}\left(h_{\theta }\left(x\right)-y\right)\\ =\left(h_{\theta }\left(x\right)-y\right)\cdot \frac{\partial }{\partial \theta_{j}}\left(\sum_{i=0}^{n}\theta_{i}x_{i}-y\right)=\left(h_{\theta }\left(x\right)-y\right)x_{j} \end{array}$$ (4)

In this way, the optimal value can be calculated, and the formula of the optimal solution can be calculated as follows:

$$\theta =\theta -\alpha \cdot \frac{\partial J\left(\theta \right)}{\partial \theta }$$ (5)

In Formula (5), α is the decreasing coefficient, and the initial value of θ can be randomly selected.

### Model Evaluation Criteria

In this paper, sensitivity (Sn), specificity (Sp), accuracy (ACC), and Matthew correlation coefficient (MCC) were still used as indicators to measure the performance of the model (Jiang et al., 2013; Wei et al., 2017a; Wei et al., 2017b; Wei et al., 2017c; Ding et al., 2019; Jin et al., 2019; Manavalan et al., 2019; Riaz and Li, 2019; Shen et al., 2019; Zeng et al., 2019a; Zeng et al., 2019b; Ding et al., 2020; Ding and JijunGuo, 2020; Hasan et al., 2020; Huang et al., 2020; Tao et al., 2020; Wan and Tan, 2020; Wang et al., 2020; Zeng et al., 2020; Zhai et al., 2020; Zhao et al., 2020; Zhang et al., 2020c; An and Yu, 2021; Ao et al., 2021a; Wang et al., 2021b; Xu et al., 2021b; Zhu et al., 2021).

$$Sn=\frac{Tp}{Tp+Fn}$$ (6)

$$Sp=\frac{Tn}{Tn+Fp}$$ (7)

$$ACC=\frac{Tp+Tn}{Tp+Tn+Fp+Fn}$$ (8)

$$MCC=\frac{Tp\times Tn-Fp\times Fn}{\sqrt{\left(Tp+Fn\right)\times \left(Tn+Fn\right)\times \left(Tp+Fp\right)\times \left(Tn+Fp\right)}}$$ (9)

Here, Tp indicates that the model correctly predicts the value of the phage virus protein; Fn represents the value of the model incorrectly predicting phage virus protein as non-phage protein; Fp represents the number of bacteriophage proteins incorrectly predicted by the model as non-phage viral proteins; and Tn indicates that the model correctly predicts the value of non-phage viral proteins.

### Summary

Phages, although very small in size, have affected our lives, not only in the environment but also in terms of our health. If a phage enters a human, it will take on a bacterial host, live in the human, and even pass on to the next generati on. This requires us to identify phages quickly and accurately, so we built a model, Pseudo-188D, to predict phage proteins. The Pseudo-188D model is roughly the overall content of Chapter 2. First, the required protein digital features were extracted by PseudoKNC software. After the lower dimensional disaster of the model AdaBoost, the features extracted by model 188D are combined with the gradient descent algorithm to predict phage virus proteins.

## Results

In this chapter, we will prove the stability and robustness of the Pseudo-188 days model from various perspectives. First, the model that we established is compared with other methods, and the stability of the model is evaluated by Sp, Sn, MCC, and Acc. Second, we used different classifiers to identify phages. By comparing the values of Sp, Sn, MCC, and Acc, it was proven that SGD was a highly correct decision for our model. Finally, we used different cross-validations to more fully prove the accuracy of our model.

### Performance Comparison of Different Characterization Methods

This section mainly proves that our model is superior to other methods and models in terms of method performance. We tried many methods to identify phage proteins, but the results were all unsatisfactory, such as those obtained with monoTriKGap (Muhammod et al., 2019), SC-PseaACC (Chou, 2005), and the 188D method for comparison. The performance of our model is stable compared with other methods. Table 1 shows the high accuracy and stability of the Pseudo-188 days model numerically, and the Sp, Sn, MCC, and Acc values are 0.89, 0.96, 0.93, and 0.85, respectively. These data indicate that the model we established is indeed suitable for phage protein identification.

**TABLE 1:** Performance comparison of different methods under 10-fold cross-validation.

Methods	Cross validation	Classification method	Sn	Sp	ACC	MCC
monoTriKGap	10-Cross validation	SGD	0.79	0.96	0.93	0.85
SC-PseAAC			0.66	0.87	0.80	0.54
188D			0.52	0.87	0.76	0.41
**Pseudo-** **188D**			**0.89**	**0.96**	**0.93**	**0.85**

### Performance Comparison of Different Classifiers

To confirm the accuracy of the classification method we selected, we compared features extracted by the PseudoKNC method at the same time, combined with features extracted by the 188D model AdaBoost with less dimensional disaster, and then verified the accuracy and stability of SGD by using 10-fold cross-validation. Finally, different classification methods were used to verify the accuracy and stability of SGD. We chose several classification methods, such as NaiveBayes (Ahmed et al., 2021), Logistic (Hosmer et al., 2015; Sikandar et al., 2019), and multilayer Perceptron (Lek and Park, 2018; Ahmad et al., 2020). Table 2 fully shows that the classification method we chose is correct. According to comparison with other methods, NaiveBayes algorithm is not stable, MCC value is only 0.49, while the ACC value is 0.94. By comparing ACC value and MCC value, it is found that the NaiveBayes classification algorithm for our model is not stable. Logistic algorithm for processing our data, Sn,Sp, ACC, MCC values are not more than 0.9, accuracy is not as high as SGD classification method; The stability of multi-layer perceptron algorithm is relatively stable, but the accuracy is 0.02 lower than SGD, so we choose SGD as the classification algorithm. Because the classifier we choose has shown its advantages, not only fast but also better accuracy than other methods.

**TABLE 2:** Performance comparison of the same method in different classifiers.

Methods	Cross validation	Classification method	Sn	Sp	ACC	MCC
Pseudo-188D	10- Cross validation	NaiveBayes	0.59	0.88	0.79	0.49
		Logistic	0.69	0.84	0.79	0.79
		Multi-layer perceptron	0.88	0.94	0.92	0.83
		**SGD**	**0.89**	**0.96**	**0.93**	**0.85**

### Performance Comparison of Different Cross-Validations

To further prove that our model can show good performance in the identification of phage protein vector features, we used Pseudo-188D processed features of the model to evaluate with different cross-validations. According to Table 3, the results of 5-fold cross-validation, 6-fold cross-validation and 8-fold cross-validation were all stable. However, it can be seen from Table 3 that when 5-fold cross-validation is selected, MCC value is 0.8, 0.05 smaller than 10-fold cross-validation, and other values are also slightly smaller than 10-fold cross-validation. When selecting the 8-fold cross-validation, the VALUE of MCC was 0.83, 0.02 smaller than the value of 10-fold cross-validation. From various indicators, the actual effect of 10-fold cross-validation was more stable and accurate than that of other methods, so 10-fold cross-validation was selected to evaluate the performance of our model.

**TABLE 3:** Performance comparison of Pseudo-188D models under different cross-validations.

Methods	Classification method	Cross validation	Sn	Sp	ACC	MCC
Pseudo-188D	SGD	5	0.86	0.94	0.91	0.80
		6	0.87	0.95	0.93	0.84
		8	0.88	0.94	0.92	0.83
		**10**	**0.89**	**0.96**	**0.93**	**0.85**

### Performance Comparison of Different Ktuple

Previously, we have introduced the influence of ktuple (k) value on the number and style of feature extraction. In this summary, we compare the accuracy and stability when k is 1,2 and 3. According to Table 4, when k value was 1, 20 feature vectors were extracted. Combined with 188 vectors extracted from 188D, the SGD classification method was used to predict phage classification, and the prediction result was 73.6156% through the performance verification of 10 fold cross validation. Not only the accuracy rate is not high, but also the stability of the classification effect is poor, the MCC value is only 0.379. When k value is 2, a total of 420 feature vectors are selected, 167 vectors are selected through model AdaBoost, and then combined with 188 feature vectors extracted from 188D, 335 feature vectors are finally selected. After selecting features, the SGD classification method was used to predict phage classification, and the performance verification of 10 fold cross validation was performed, and the prediction result was 78.5016%. The prediction result obtained was far better than the final result of our model Pseudo-188D, and the MCC value was only 0.503, so we did not choose k values of 1 and 2.

**TABLE 4:** Performance comparison under different Ktuple (k).

Ktuple (k)	Dimension	Sn	Sp	ACC	MCC
1	208	0.54	0.83	0.74	0.379
2	335	0.65	0.85	0.78	0.503
**3**	**448**	**0.89**	**0.96**	**0.93**	**0.85**

### Summary

In this chapter, we have compared the monoTriKGap, SC-PSEaAC, and 188D methods and different classification methods. We have also compared different cross-validations, and our model Pseudo-188D shows good performance. To demonstrate Pseudo-188D performance more clearly, we combined the phage proteins extracted by the PseudoKNC method with the features extracted by 188D after AdaBoost treatment of the model. Then, 80% of feature vectors were randomly selected as the test set, and the training model and the remaining 20% of feature vectors were selected as the test set to verify the robustness of our model. The experimental results show that the model pseudo-188 days still shows good performance, and the accuracy of the results reaches 95.082%. Moreover, the values of Sp, Sn, MCC, and Acc also show good stability, reaching 0.94, 0.93, 0.95, and 0.89, respectively. These values fully demonstrate the stability and accuracy of Pseudo-188D.

Phages affect human lives all the time, and some of them are latent and inherited in the human body. Phages can also be used if they are understood. Many years ago, phages successfully prevented *Pseudomonas aeruginosa* infection in burn patients. Therefore, we need to accurately identify phages so that they can be used for medical research or prevention and control of life inconveniences caused by phages. When establishing the model in this paper, we choose PseudoKNC to extract features. When the k value is 3, a total of 8420-dimensional features are extracted. After processing the AdaBoost model, 260 features with the best performance are selected, and combined with features extracted from 188D, there are 448-dimensional features. The 448-dimensional vectors were classified by SGD, and the accuracy was 93.4853% under 10-fold cross verification. To further improve the rigor of the experiment, we randomly selected 80% of the data as the test set and the remaining 20% as the validation set. After this validation, our model pseudo-188 days still showed stability and accuracy and, most importantly, significantly saved time and cost.

## Conclusion

This paper mainly introduces the Pseudo-188D model that we established, which accurately predicts phage proteins and makes a small contribution to phage prediction, improving the accuracy of phage prediction. In addition, our model greatly reduces the time and expense of predicting phage proteins, which saves considerable time and money. The greatest innovation in this paper is the combination of PseudoKNC and 188D, which can improve the predictive accuracy of phages. This will facilitate phage research, whether it is using phages for medical problems, anticancer methods based on phages, or solving environmental problems around us. That is where the value of phage research is realized.

## Data Availability

The original contributions presented in the study are included in the article/Supplementary Material, further inquiries can be directed to the corresponding author.
